# Characterization of chronic HCV infection-induced apoptosis

**DOI:** 10.1186/1476-5926-10-4

**Published:** 2011-07-23

**Authors:** Abdel-Rahman N Zekri, Abeer A Bahnassy, Mohamed M Hafez, Zeinab K Hassan, Mahmoud Kamel, Samah A Loutfy, Ghada M Sherif, Abdel-Rahman El-Zayadi, Sayed S Daoud

**Affiliations:** 1 Virology and Immunology Unit, Cancer Biology Department, National Cancer Institute, Cairo University, Egypt; 2 Pathology Department, National Cancer Institute, Cairo University, Egypt; 3 Clinical Pathology Department, National Cancer Institute, Cairo University, Egypt; 4 Biostatistic & Epidemiology Department, National Cancer Institute, Cairo University, Egypt; 5 Tropical Medicine Department, Ain Shams University, Egypt; 6 Center for Integrated Biotechnology, Washington State University, Pullman, WA, USA

## Abstract

**Background:**

To understand the complex and largely not well-understood apoptotic pathway and immune system evasion mechanisms in hepatitis C virus (HCV)-associated hepatocellular carcinoma (HCC) and HCV associated chronic hepatitis (CH), we studied the expression patterns of a number of pro-apoptotic and anti-apoptotic genes (Fas, FasL, Bcl-2, Bcl-xL and Bak) in HepG2 cell line harboring HCV- genotype-4 replication. For confirmation, we also assessed the expression levels of the same group of genes in clinical samples obtained from 35 HCC and 34 CH patients.

**Methods:**

Viral replication was assessed in the tissue culture medium by RT-PCR, quantitative Real-Time PCR (qRT-PCR); detection of HCV core protein by western blot and inhibition of HCV replication with siRNA. The expression level of Fas, FasL, Bcl-2, Bcl-xL and Bak was assessed by immunohistochemistry and RT-PCR whereas caspases 3, 8 and 9 were assessed by colorimetric assay kits up to 135 days post infection.

**Results:**

There was a consistent increase in apoptotic activity for the first 4 weeks post-CV infection followed by a consistent decrease up to the end of the experiment. The concordance between the changes in the expression levels of Fas, FasL, Bcl-2, Bcl-xL and Bak *in vitro *and *in situ *was statistically significant (p < 0.05). Fas was highly expressed at early stages of infection in cell lines and in normal control liver tissues followed by a dramatic reduction post-HCV infection and an increase in the expression level of FasL post HCV infection. The effect of HCV infection on other apoptotic proteins started very early post-infection, suggesting that hepatitis C modulating apoptosis by modulating intracellular pro-apoptotic signals.

**Conclusions:**

Chronic HCV infection differently modulates the apoptotic machinery during the course of infection, where the virus induces apoptosis early in the course of infection, and as the disease progresses apoptosis is modulated. This study could open a new opportunity for understanding the various signaling of apoptosis and in the developing a targeted therapy to inhibit viral persistence and HCC development.

## Background

Hepatitis C virus (HCV) is a major worldwide causative pathogen of chronic hepatitis, cirrhosis, and hepatocellular carcinoma [1]. Egypt has the highest prevalence of HCV infection in the world where 15% of the total population are infected [2-4]. Although the exact mechanisms of HCV pathogenesis, such as viral persistence, hepatocytes injury, and hepatocarcinogenesis are not fully understood, yet an accumulating body of evidence suggests that apoptosis of hepatocytes is significantly involved in the pathogenesis [5,6].

Apoptosis plays a pivotal role in the maintenance of cellular homeostasis through removal of aged cells, damaged cells, and overgrowing new cells [7]. Failure of apoptosis induced by various stimuli is one of the most important events in tumor progression as well as in resistance to cytotoxic therapy [8]. In mammalian cells, apoptosis can be induced via two major pathways. First, the death receptor pathway (extrinsic pathway), which is triggered by binding Fas ligand (FasL) to Fas (CD95) with subsequent activation of caspase-8, which in turn activates the effectors caspases 3, 6, 7 [9-12]. This pathway is considered an important apoptotic system in cancer [13] because FasL is one of the effector molecules of cytotoxic T cells. The second apoptosis pathway (the intrinsic pathway) is induced by mitochondria in response to DNA damage, oxidative stress and viral proteins [5]. Mitochondria-dependent apoptosis is amplified by pro-apoptotic genes (Bax, Bad, Bak and others) whereas molecules like Bcl-2 or Bcl-xL act as anti-apoptotic. These proteins converge at the mitochondrial permeability transition pore that regulates the release of apoptotic regulatory proteins, such as procaspase-9, and cytochrome C [14].

There have been many studies indicating that apoptosis of hepatocytes plays a significant role in the pathogenesis of HCV infection [15], although various apoptotic pathways were proposed [16]. For example, many studies demonstrated that HCV core protein suppresses apoptosis mediated by cisplatin, c-myc, TNF-α, or the Fas signaling pathway [17], whereas others showed that the core protein sensitizes Fas, TNFα, or serum starvation-induced apoptosis [18]. The precise mechanisms for the involvement of the HCV core protein on the apoptotic pathways are not fully understood. For example, core protein-dependent inhibition of TNF-α and CD95 ligand-induced apoptosis has been described in a hepatoma cell line [19,20]. In other models, overexpressed HCV core protein did not prevent CD95 ligand induced apoptosis in hepatoma cells or transgenic mice overexpressing HCV core protein [17,21]. Until recently, the lack of an infectious HCV tissue culture system did not allow to study the impact of HCV infection on hepatocyte apoptosis [22].

The present study was performed to determine the changes in apoptotic machinery accompanying HCV infection both *in vitro *and *in vivo*. For the *in vitro *study, we developed a HCV replication system in HepG2 cell line, which may reflect to some extent the *in vivo *situation. Successful infection and propagation of the virus was assessed by detection of HCV-RNA using nested RT-PCR with specific primers, detection of increased titer by real time PCR, and virus passage to naïve cells. The HCV-HepG2 cell line was then used to study the long term effect of HCV infection on the apoptosis regulatory genes (Fas, FasL, Bak, Bcl-2, and Bcl-xL). This was correlated with the apoptotic activity in the cells by determining the expression levels of caspases 3, 8, and 9. We further assessed protein expression and mRNA levels of the same group of genes in liver tissues tissue samples obtained from patients with chronic hepatitis (CH) and hepatocellular carcinoma (HCC).

## Methods

### Patients

The present study included 69 cases that are HCV-RT-PCR positive and HBV-PCR negative in both liver tissues and serum samples. These cases were divided into two groups: group 1 (HCC; *n *= 35), samples were collected from patients diagnosed and treated at the National Cancer Institute, Cairo University, between December 2005 and August 2008; group 2 (CH; *n *= 34), samples were collected from HCV associated chronic hepatitis (CH) patients admitted to Kasr Al-Aini School of Medicine, Cairo University, in the same period and enrolled in routine diagnosis or therapeutic procedures. The mean age of CH patients was 47.5 years and M:F ratio was 1.5:1, whereas the mean age of HCC was 51.6 years and M:F ratio was 1.3:1.

All cases of CH were graded and staged according to the modified Knodell scoring system [23] and all HCC cases were graded according to the World Health Organization (WHO) classification criteria and staged according to the American Joint Committee on Cancer [24]. The percent of normal to tumor ratio were more than 80% in all studied cases to overcome the nominalization effect of the tumor stroma and/or necrosis as well as the cirrhotic tissues factors in the studied specimens. Table 1 illustrates the clinico-pathological features of the studied cases. Normal liver tissue samples were obtained from liver transplant donors (15 samples) and were used as controls. A written consent was obtained from all patients and normal liver donors prior to enrollment in the study and the ethical committee of NCI approved the protocol, which was in accordance with the ethical guidelines of the 1975 Declaration of Helsinki.

**Table 1:** Clinical features of the studied groups of patients.

Variables	HCC	CH
	
	*n *= 35 (%)	*n *= 34 (%)
Liver Function Test (Mean ± SD)		
ALT	77.2 ± 76.2	74.33 ± 30.97
AST	70.577 ± 49.4	81.66 ± 35.35
Alk ph	181.1 ± 174.2	111.57 ± 61.58
Alb	3.758 ± 0.707	3.9 ± 0.538
T.Bil	1.1846 ± 0.523	1.34 ± 0.897
INR	1.179 ± 0.067	1.22 ± 0.161

Complete Blood Picture (Mean ± SD)		
Hb	12.3 ± 1.64	13.59 ± 2.24
TLC	6.186 ± 3.163	6.509 ± 2.05
Plt	177 ± 121	175.5 ± 67.267
Viral marker		
HBs-Ag	0 (0)	0 (0)
HCV-Ab	35 (100)	34 (100)
HBV-PCR	0 (0)	0 (0)
HCV-PCR	35 (100)	34 (100)

Tumor Marker (Mean ± SD)		
Serum AFP	1885 *± *5888	265 *± *110

AFP, alpha fetoprotein; Alb, albumin; Alk, Alkaline Phosphates; ALT, alanine aminotransferase; CH, chronic hepatitis; Hb, hemoglobin; HBs-Ag, hepatitis B surface antigen; HCC, hepatocellular carcinoma; HCV, hepatitis C virus; INR, International normalized ratio; PCR, polymerase chain reaction; Plt, platelet count; TLC, total leukocytic count; T.Bil, total bilirubin.

### HepG2 cell culture

HepG2 cells were used to establish the *in vitro *HCV replication. HepG2 culturing and infection were carried out according to previous protocols [25]. Briefly, HepG2 cells were maintained in 75 cm culture flasks (Greiner bio-one GmbH, Germany) containing Dulbecco's Modified Eagle's Medium (DMEM) supplemented with 4.5 g/L glucose and 10 g/L L-glutamine (Bio Whittaker, a Combrex Company, Belgium), 50 ml/L fetal calf serum (FCS), 10 g/L penicillin/streptomycin and 1 g/L fungizone (250 mg/L, Gibco-BRL life Technologies, Grand Island, NY (USA). The complete culture medium (CCM) was renewed every 3 days, and cells were passaged every 6-10 days. A total of 3 × 10^6 ^cells were suspended in 10 ml CCM and incubated at 37°C in 5% CO_2_.

### Viral inoculation and sample collection

Viral inoculation and cell culture were performed as previously described [26]. Briefly, cells were grown for 48 h to semi-confluence in complete culture medium, washed twice with FCS-free medium, and then inoculated with 500 μl serum obtained from HCV infected patients (500 μl patient sera and 500 μl FCS-free DMEM/3 × 10^6 ^cells). The HCV genotype was characterized as genotype-4 with 9 quasispecies based on our previously described method [27]. The viral load in the used serum was quantified by real time PCR. The average copy number was 58 × 10^7^copies/ml. After 180 min, Ham F12 medium (Bio Whittaker, a Combrex Company, Belgium) containing FCS was added to make the overall serum content 100 ml/L in a final volume of 10 ml including the volume of the human serum, which used for infection as mentioned above. Cells were maintained overnight at 37°C in 5% CO_2_. The next day, adherent cells were washed with CCM and incubation was continued in CCM with 100 ml/L FCS. Throughout the culture duration, the assessment of HCV replication were confirmed by a detection of viral core protein using western blotting, by RT-PCR amplification of sense and antisense strands of the virus by real time PCR and by the inhibition of HCV replication using siRNA knockout as we previously reported [28].

### Western blot analysis of HCV core antigens in HepG2 cells

Lysates containing 100 μg of protein from uninfected and infected HepG2 cells were subjected to SDS-PAGE, as previously described [26,27]. After three washes, membranes were incubated with diluted peroxidase-labeled anti-human IgG/IgM antibody mixture at 1:5000 in PBS (3 g/L) for previously treated strips with the anti-core antibody (Novocastra, Novocastra Laboratories, UK) for 2 h at room temperature. Visualization of immune complexes on the nitrocellulose membranes was performed by developing the strips with 0.01 mol/L PBS (pH 7.4) containing 40 mg 3,3',5,5'-tretramethylbenzidine and 100 μl of 30 ml/L hydrogen peroxide (Immunopure TMB substrate Kit, PIERCE, Rockford, IIIinois, USA).

### Quantification of human GAPDH mRNA

The integrity of the cellular RNA preparations from HCV infected HepG2 cells was analyzed by 18s and 28s bands on agarose gel and by automated gel electrophoresis (Experion Software Version 3.0, Bio-Rad), which was also used for measuring the RNA concentration in addition to spectrophotometer at 260 nm (nanoDrop, USA). GAPDH mRNA levels were quantified by real time RT-PCR using TaqMan technology with GAPDH specific primers. Amplification of human GAPDH transcripts was performed using the TaqMan EZ RT-PCR kit (Applied Biosystems, Foster City, CA). The target template was the purified cellular RNA from HepG2 cells at 1, 2, 3, 4, 5, 6, 7 and 8 days post-infection with HCV, in absence and presence of siRNA. The RT-PCR was performed using a single-tube, single-enzyme system. The reaction exploits the 5'-nuclease activity of the rTth DNA polymerase to cleave a TaqMan fluorogenic probe that anneals to the cDNA during PCR 50 μl reaction volume, 1.5 μl of RNA template solution equivalent to total cellular RNA from 2.5 × 10^5 ^cells were mixed with 200 nM forward primer, 200 nM reverse primer, 300 nM GAPDH probe, 300 μM from each of dATP, dCTP, dGTP and 600 μM dUTP, 3 mM manganese acetate, 0.5 μl rTth DNA polymerase, 0.5 μl Amp Erase UNG, 1× Taqman EZ buffer and amplified in the sequence detection system ABI 7700 (Applied Biosystems, Foster City, CA). The RT-PCR thermal protocol was as follows: Initial UNG treatment at 50°C for 2 minutes, RT at 60°C for 30 minutes, deactivation of UNG at 95°C for 5 minutes followed by 40 cycles, each of which consists of denaturation at 94°C for 20 seconds and annealing/extension at 62°C for 1 min.

### Northern Blot Analysis

To construct a HCV RNA transcription vector total RNA was extracted from all cell types at days 1, 2, 3, 4, 5, 6, 7 and 8 post-transfection, 5 μg of total RNA were loaded onto the gel. HCV sequences from nt 47 to 1032 were cloned after RT-PCR into pSP 64 [poly(A)] vector (Promega), resulting in plasmid PMOZ.1.HCV then confirmed by DNA sequence analysis. HCV template RNA was transcribed *in vitro *from MOZ.1.HCV. Briefly, 5 mg of plasmid DNA was linearized with a BglII. The linear plasmid DNA was purified from an agarose gel and then incubated with 50 U of SP6 RNA polymerase for 2 h at 37°C in the presence of 500 mM (each) ribonucleoside triphosphates (GTP, ATP, UTP, and CTP), 100 U of RNAsin, 10 mM dithiothreitol, 40 mM Tris-HCl (pH 7.5), 6 mM MgCl2, 2 mM spermidine, and 10 mM NaCl in a total reaction volume of 100 μl. After transcription reaction, DNA template was degraded by two rounds of digestion with RNase-free DNase (Boehringer) for 30 min at 37°C with 10 U of enzyme. Upon completion of digestion, two rounds of extraction with phenol-chloroform-isopropyl alcohol and then ethanol precipitation were done. HCV RNA transcripts, which contained a poly(A) tail, were further purified on an oligo(dT) cellulose column. RNA concentration was determined spectrophotometrically at A260 with UV light. An aliquot was analyzed by agarose gel electrophoresis to assess its integrity.

### Sensitivity of RT-PCR assay

HCV RNA synthesized *in vitro *was diluted with TE (Tris-EDTA) buffer at a concentration of approximately 106 copies per ml and was stored at -20°C. Serial 10-fold dilutions of these stock solutions were made in water just prior to RT-PCRs. One hundred copies were routinely detected. Both probes were purified using MicroSpin G-50 columns (Amersham Pharmacia). Blots were visualized and quantified as previously described [29].

### Detection of plus and minus-strand RNA by nested RT-PCR

Detection of plus- and minus- HCV strand was performed as previously reported [26,30]. The One Step real-time PCR system (Applied Biosystems) was used.

### Molecular detection of HBV

DNA extraction and PCR amplification from fresh tissues and PCR amplification were performed as previously described [31].

### Determination of caspase activity

HepG2 cells were harvested on different dates. After lysis and protein concentration, cell lysates containing 200 μg of total protein was used to measure the activities of caspases 3, 8 and 9 using ApoTaget colorimetric Assay kits (BioSource international, Inc. Camarillo, CA) according to the manufacturer instructions.

### RNA extraction from liver tissues

Total RNAs were extracted using a SV total RNA isolation system (Promega, Biotech) according to manufacturer's instructions. The extracted total RNA was assessed for degradation, purity and DNA contamination by a spectrophotometer and electrophoresis in an ethidium bromide-stained 1.0% agarose gel. Ten samples of normal human DNA and RNA were extracted from normal liver tissues and were used to optimize the best conditions for the multiplex PCR of *B-actin *gene (621-bp fragments) *versus *each of the studied genes. Negative RT-PCR control was used against each sample [32].

### c-DNA synthesis

Reverse transcription (RT) of the isolated total RNA was performed in 25 μl reaction volume containing 200 u of Superscript II RT enzyme (Gibco-BRL, Gaithersburg, MD, USA.), 1× RT-buffer [250 mM Tris-HCl pH 8.3, 375 mM KCl, 15 mM MgCl2], 1 mM dithiotheritol, 25 ng from random primer, 0.6 mM deoxynucleotide triphosphates, 20 U RNAsin (Promega, USA.), 100 ng of extracted RNA. Samples were then incubated at 50°C for 60 min followed by 4°C until the PCR amplification reaction [32].

### PCR amplification of the studied genes

Primer sequences, PCR conditions of the studied genes (Fas, FasL, Bcl-2, Bcl-xL and Bak), and the expected PCR DNA band length are listed in Table 2. The PCR and quantitation were performed in a 50 μL reaction volume containing 5 μL of the RT reaction mixture (c-DNA), 2.5 units Taq polymerase (Gibco-BRL, Gaithersburg, MD, USA), 1× PCR buffer (500 mM KCl, 200 mM Tris-HCl, 1.5 mM MgCl_2_, 1 mg/mL bovine serum albumin (BSA)), 200 mM each of the deoxyribonucleotide triphosphate and 0.25 mM of each primer. Amplification of the β-actin gene (621 bp fragment) was performed to test for the presence of artifacts and to assess the quality of RNA. A water control tube containing all reagents except c-DNA was also included in each batch of PCR assays to monitor contamination of genomic DNA in the PCR reagents. Negative RT-PCR control was used against each sample [32].

**Table 2:** Primer sequences of the studied genes.

Gene Name	Primer Sequence	Fragment Length
**β-actin**	5'-ACA CTG TGC CCA ACG AGG-3' 5'-AGG GGC CGG TCA T AC T-3	621 bp
**Fas**	5'-GCAACACCAAGTGCAAAGAGG-3' 5'-GTCACTAGTAATGTCCTTGAGG-3'	265 bp
**FasL**	5'- ATGTTTCAGCTCTTCCACCTACAGA-3' 5'-CCAGAGAGAGCTCAGATACGTTGAC-3'	255 bp
**Bak**	5'-TGATACCTGTGCTTTATCCC -3' 5'- AAACCAGCATCTCTCTAAAC-3'	250 bp
**Bcl-2**	5' GCAGATCCAGGTGATTCTCG 3' 5' ATCGATGCCAATGACAGCCA 3'	234 bp
**Bcl-XL**	5'-CCCGGTGCTGCAGCATGTCCT -3' 5'-TCCCCTCGAGGATTTCGACAG -3'	521 bp

### Quantification of the studied genes

Fifteen microliters of each PCR product were separated by electrophoresis through a 2.0% ethidium bromide-stained agarose gel and visualized with ultraviolet light. Gels were photographed and the bands were scanned as digital peaks. Areas of the peaks were then calculated in arbitrary units with a digital imaging system (Photo-documentation system, Model IS-1000; Alpha Innotech Co., San Leandro, CA, USA). To evaluate the relative expression levels of target genes in the RT-PCR, the expression value of the normal pooled liver tissues was used as a normalizing factor and a relative value was calculated for each target gene amplified in the reaction. Non-expression in any of the studied genes was considered if there was a complete absence, or more than a 75% decrease in the intensity of the desired band in comparison to the band of normal pooled liver tissue [24,25]. Samples were assayed in batches that included both cases and controls. The absence of bands was confirmed by repeating the RT-PCR twice at different days and by consistent presence of β-actin gene amplification [32].

### Immunohistochemistry

Protein expression of the studied proteins was assessed using the following monoclonal antibodies Fas (C236), FasL (sc-56103), Bcl-2 (sc-56016), and Bcl-xL (sc-8392) (all from Santa Cruz Biotechnology, inc. Germany). Briefly, from each tumor block, a hematoxylin and eosin-stained slide was microscopically examined to confirm the diagnosis and select representative tumor areas. Tissue cores with a diameter of 1.5 mm were punched from the original block and arrayed in triplicate on 2 recipient paraffin blocks. Five μm sections of these tissue array blocks were cut and placed on positive charged slides to be used for IHC analysis. Sections from tissue microarrays were deparaffinized, re-hydrated through a series of graded alcohols, and processed using the avidin-biotin immunoperoxidase methods. Diamino-benzidine was used as a chromogen and Mayer hematoxylin as a nuclear counterstain. A case of follicular lymphoma was used as a positive control for Bcl-2, Fas and FasL whereas a case of colon cancer was used as a control for Bcl-xL. Results were scored by estimating the percentage of tumor cells showing characteristic cytoplasmic immunostaining for all examined markers [33].

Protein expression was classified compared to normal hepatic tissue samples. Positive expression was further classified according to the level of expression into mild: ≥ 10%- < 25%, moderate: ≥ 25%- < 50% and high expression: ≥ 50% but during statistical analysis they were broadly classified into negative or positive expression.

### Statistical analysis

The results were analyzed using the Graph Pad Prism software (Graph Pad Software, San Diego, CA, USA). For gene expression analysis the Mann-Whitney U Test was used for numeric variables and Chi square or Fisher's exact Test were used to analyze categorical variables. *P*-value was considered significant when ≤ 0.05.

## Results

All studied cases were positive for HCV infection by both ELISA and HCV RT-PCR in serum and liver tissue but were negative for HBV infection by serological markers and PCR both in serum and liver tissues. The level of pro-apoptotic genes expression was measured in HCV infected HepG2 cell line as an *in vitro *model as well as in HCC and CH tissue samples.

### Infection of HepG2 cell line with hepatitis C virus

**Figure 1:**
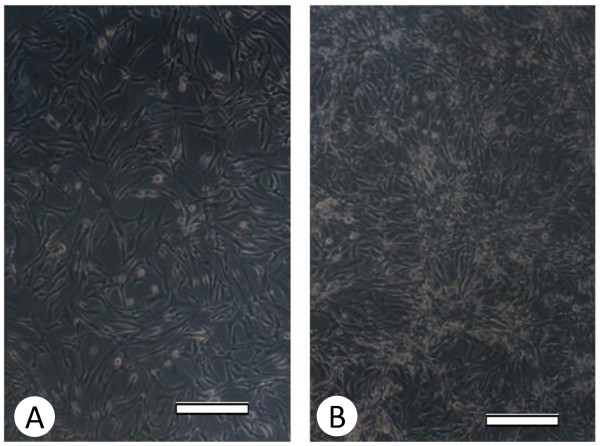
**(A): Non-infected HePG2 cells**. (B): Infected HePG2 cells. Scale bar = 100 μm.

**Figure 2:**
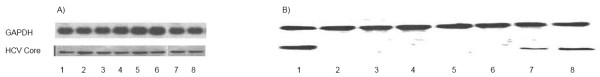
**Expression levels of the viral core and GAPDH**. (A) The expression level of the viral core and GAPDH in HepG2 cells infected by HCV genotype-4 from day 1 to day 8. (B) The expression level of the viral core in HepG-2 cells infected by HCV genotype-4 from day 1 to day 8. Upper row show HCV-core expression in un-transfected cells. Lower row showed the HCV- core expression in siRNA-Z5 transfected cells.

In this model, we observed a good correlation between persistence of HCV infection in HepG2 cell line and the appearance of certain morphological changes in the infected cells such as visible cell aggregation and granulation that took place 21 days post infection suggesting successful viral transfection, as shown in Figure 1. Successful HCV genotype-4 replication in HepG2 cells were also confirmed by western blot for the detection of viral core protein as shown in Figure 2a, as well as inhibition of HCV replication by 100 nM siRNA previously developed in our lab [28], illustrated in Figure 2b.

Quantification of HCV RNA was performed both in cell free media and cell lysates at days 1, 2, 3, 7, 14, 21, 28, 35, 42, 52, 59 and 116 post HCV infection. HCV RNA was detected in all of these days except days 35, 52 for cell free media and days 21, 28 for cell lysates. HCV-RNA was quantitatively detected in all days except days 2, 3, 14, 45 (Table 3).

**Table 3:** Changes in apoptotic and pre apoptotic genes expression in HCV infected HepG2 cell line *in vitro*.

	Qualitative/Quantitative PCR (copy number/ml)		Apoptotic gene				
**Days**	**Cell free media**	**Cell lysate**	**Bcl-xL**	**Bcl-2**	**Bak**	**Fas**	**FasL**

Day1	Positive/785	Positive	-	+	+++	*++*	-
Day2	Positive/Negative	Positive	-	+	+	*++*	-
Day3	Negative/Negative	Positive	-	+	++	*++*	-
Day7	Positive/13005	Positive	-	+	+	-	-
Day14	Positive/Negative	Positive	-	+	+	-	-
Day21	Negative/6782	Positive	-	+	-	-	*+*
Day28	Negative/24678	Positive	+	+	++	-	*+*
Day35	Positive/8892	Negative	-	+	-	-	*+*
Day45	Positive/Negative	Positive	+	+	-	-	*++*
Day52	Positive/7374	Negative	-	+	-	-	*+++*
Day59	Positive/22963	Positive	+	+	++	-	*+++*
HepG2 Control	-	-	-	+	+	*+*	-

+: Equal to the expression level in the HepG2; ++: twofold increase in the expression level; +++ threefold increase in the expression level.

### Apoptotic genes expression in HCV-infected HepG2 cells

**Figure 3:**
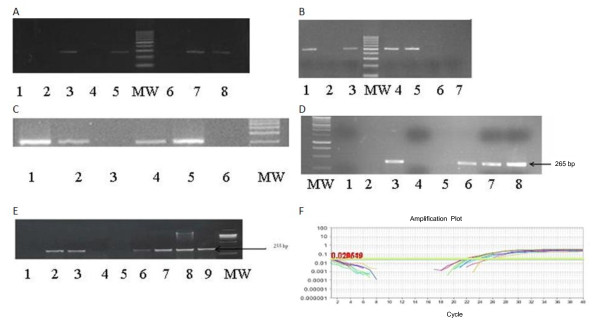
**Data on gene amplification**. Ethidium bromide-stained 2% agarose gel (A) for *Bcl2 *gene amplification. Lanes 1 and 2 showed negative RT-PCR control; lane 3 showed positive amplification of CH case; lane 4 showed negative amplification of CH case; lane 5 showed positive amplification of HCC case; lane 6 showed negative amplification of HCC case; lane 7 showed positive amplification of HepG2 without HCV infection; lane 8 showed positive amplification of HepG2 with HCV infection. (B) For *Bcl-Xl *gene amplification. Lane 1 showed HepG2-positive amplification with HCV infection at day 28; lane 2 HepG2-negative amplification without HCV infection; lane 3 and 4 showed positive amplification of CH case; lane 5 showed positive amplification of HCC case; lane 6 & 7 showed negative RT-PCR control. (C) For Bak gene amplification. lane 1 HepG2-positive amplification with HCV infection at days 59; lane 2 HepG2-negative amplification without HCV infection lane 3 showed HepG2-negative amplification with HCV infection at days 35; lane 4 showed positive amplification of CH case; lane 5 showed positive amplification of HCC case of CH; lane 6 negative RT-PCR control. (D) for *Fas *gene amplification, first lane: MW, lanes 1 and 2: negative RT-PCR control, lane 3 showed HepG2-positive amplification without HCV infection, lane 4 HepG2- showed negative amplification with HCV infection at day 21, lane 5 showed negative case of HCC, lanes 6 and 7 showed positive amplification of CH and lane 8 showed positive amplification of HCC case. (E) for *FasL *gene amplification, lane 1: negative RT-PCR control; lanes 2 and 3 showed HepG2-positive amplification with HCV infection at days 28 and 35 respectively; lane 4 showed HepG2-negative amplification without HCV infection; lane 5 showed negative case of CH; lanes 6 and 7 showed positive amplification of CH, lanes 8 and 9 showed positive amplification of HCC case. (F) Amplification plot of RT-PCR for housekeeping gene using Taqman probe.

No changes in the expression level of Bcl-2 gene post-HCV infection was observed compared to the control (HCV free HepG2 cells) (Figure 3A). The expression of Bcl-xL and Bak genes (Figures 3B, C, respectively) fluctuated 3 weeks post infection then, the levels of their expression was similar to the control levels at the end of the experiment. Interestingly, there was a good correlation between Fas, FasL genes expression and HCV infection. The expression of Fas gene was visible until the third measurement (day 3) post infection and then disappeared by the end of the experiment. In contrast, the expression of FasL was not visible until day 21 post infection then the visibility progressively increased until the end of the experiment (Table 3 Figures 3D, E).

### Caspases activity in HCV-infected HepG2 cells

**Figure 4:**
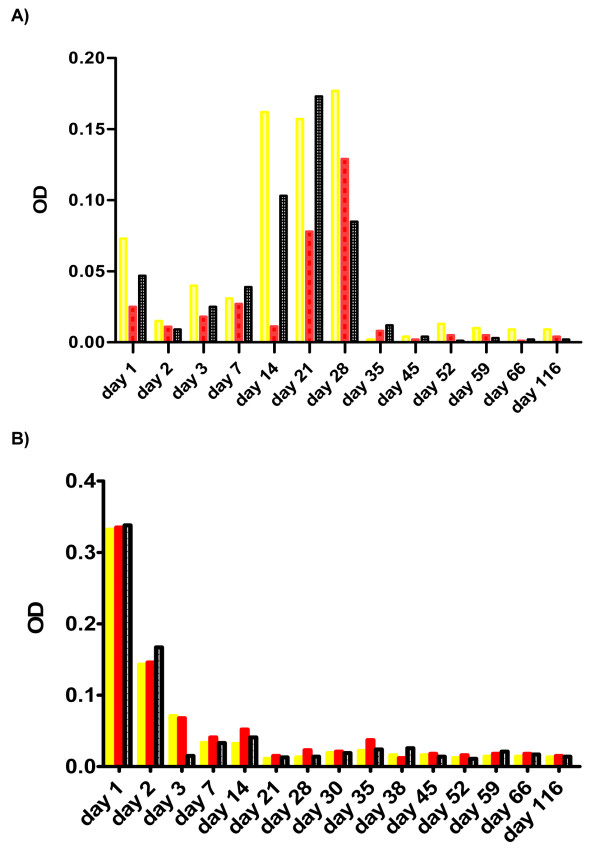
**Changes in caspases expression levels *in vitro***.

As shown in Figure 4, recognizable changes were observed in caspases 3, 8 and 9 throughout the course of HCV infection. There was an initial increase in their levels starting from day six to day 30 then all caspases levels were dramatically decreased until day 135 post-infection.

### Apoptotic genes expression in the studied cohorts of patients

**Figure 5:**
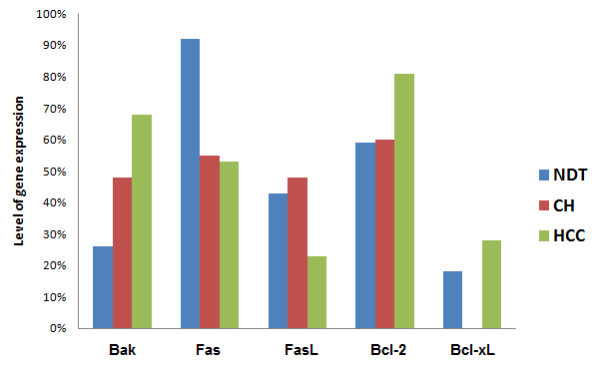
**The expression level of the apoptotic genes in the different studied groups**. NB: CH = Chronic hepatitis, HCC = Hepatocelullar carcinoma, NAT = Normal distant to tumor.

There was a significant difference in the RNA expression level of both Bcl-xL and Bcl-2 genes between HCC and CH (26%, 80% *versus *0%, 59%; respectively, p < 0.0001, = 0.0068). As well as between HCC cases and normal distant tumor (NDT) (p < 0.001) (Figure 5). Similarly, a significant difference was found in the Bak gene expression between HCC and CH patients (69% *versus *47%, p = 0.0025) as well as between HCC and NDT (p < 0.0001). The FasL was significantly expressed in CH compared to HCC (47% *versus *23%, p < 0.001). None of the CH cases studied revealed Bcl-xL gene expression.

### Apoptotic proteins expression

**Figure 6:**
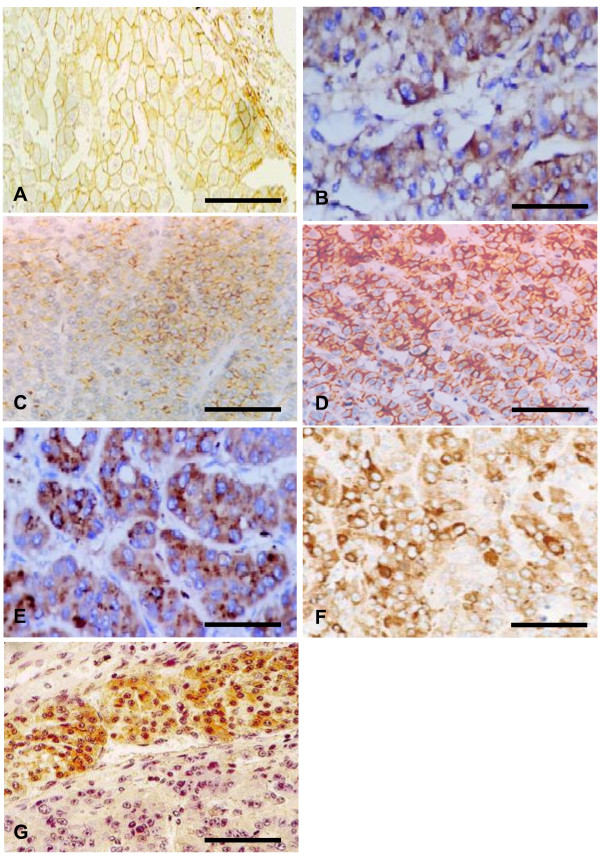
**Cases of chronic hepatitis (CH) and hepatocellular carcinoma (HCC)**. Data from cases of CH showing (A) high membranous expression of FasL, (B) moderate cytoplasmic expression of FAS and (C) moderate cytoplasmic expression of Bcl-2. Cases of HCC showing (D) High membranous expression of FasL, (E) Marked expression of FAS, (F) high expression of Bcl-2, and (G) Marked expression of Bcl2 in tumor tissues with loss of expression in adjacent non neoplastic region. Scale bar = 100 μm (A, C, D, G) and 200 μm (B, E, F).

Positive immunostaining for Bcl-2, Bcl-xL, Fas and FasL proteins was detected in 29 (85.9%), 12 (34.3%), 21 (60%) and 9 (25.7%) the studied samples of the 35 HCC cases examined compared to 18 (52.9%), 0 (0%), 18 (52.9%) and 18 (52.9%) of samples of the 34 CH cases; respectively. The concordance between immunohistochemistry and RT-PCR ranged from 86% to 94% (Figure 6).

### Clinical correlations

In HCC cases, Fas-RNA and protein expression were significantly associated with the presence of cirrhosis (p = 0.0027) and with poorly differentiated tumors (p < 0.0001). Bak gene expression was significantly associated with the presence of invasion (p = 0.05), absence of cirrhosis (p < 0.0001) and with well differentiated tumors (p < 0.0001). The expression level of Bcl-2-RNA and protein was significantly associated with poorly differentiated tumors (p < 0.0001) (Table 4).

**Table 4:** Correlation between gene expression and clinicopathological features in hepatocellular carcinoma cases.

Variable N = 35 (%)	Bak N = 24 (%)	Fas N = 19 (%)	FasL N = 8 (%)	Bcl-2 N = 28 (%)	Bcl-xL N = 9 (%)
**Age (mean ± SD)**					
57 ± 10.2					
≤ 55: 16 (46)	12 (75)	7 (44)	3 (19)	10 (63)	5 (31)
> 55: 19 (54)	12 (63)	12 (63) #	5 (26)	18 (95) #	4 (21)
**Gender**					
M: 22 (63)	17 (77) #	12 (55)	5 (23)	19 (86) #	4 (18)
F: 13 (37)	7 (54)	7 (54)	3 (23)	9 (69)	5 (38) #
**Tumor size**					
≤ 8: (22)	14 (64)	10 (45)	4 (18)	17 (77)	5 (23)
> 8: (13)	10 (77)	9 (69)	5 (38)	9 (69)	4 (31)
**Grade**					
II: 22 (63)	16 (73) #	10 (45)	4 (18)	17 (77)	6 (27)
III: 13 (37)	8 (61)	9 (69) #	4 (31)	11 (84) #	3(23)
**Invasion**					
Positive: (18)	15 (83) #	12 (67)	6 (33)	15 (83)	6 (33)
Negative: (17)	9 (53)	7 (41)	2 (12)	13 (76)	3 (18)
**Cirrhosis**					
Present: 15 (43)	8 (53)	10 (67) #	4 (27)	12 (80%)	3 (20)
Absent: 20 (57)	16(80) #	9 (45)	4 (20)	16 (80%)	6 (30)

# significantly difference (p < 0.005)

Table 5 shows that in CH patients Fas expression was significantly associated with high hepatitis grade (p = 0.05), whereas FasL expression was significantly associated with the presence of necrosis as well as with high hepatitis grade and stage (p = 0.015, 0.015 and 0.006; respectively). In contrast, Bcl-2 expression was significantly associated with the presence of cirrhosis (p < 0.0001).

**Table 5:** Correlation between gene expression and clinicopathological features in CH patients

Variable N = 34 (%)	Bak N = 16 (%)	Fas N = 19 (%)	FasL N = 16 (%)	Bcl-2 N = 20 (%)
**Age (mean ± SD)**				
44 ± 9.8				
≤ 47: 18 (53)	8 (44)	13 (72) #	8 (44)	9 (50)
> 47: 16 (47)	8 (50)	6 (38)	8 (50)	11 (69)
**Gender**				
M: 31 (91)	13 (41)	17 (55)	15 (48)	18 (58)
F: 3 (8)	3 (100) #	2 (67) #	1 (33)	2 (66)
**Steatosis**				
Absent: (10)	3 (30)	3 (30)	2 (20)	4 (40)
Minimal: (14)	7 (50)	9 (64)	4 (29)	10 (71)
Moderate: (7)	4 (57)	5 (71)	7 (100)	5 (71)
Marked: (3)	2 (67)	2 (67)	3 (100)	1 (33)
**Necrosis**				
Absent: (26)	12 (46)	13 (50)	9 (35)	16 (62)
Minimal: (8)	4 (50)	6 (75)	7 (88) #	4 (50)
**Necro-inflammation**				
Absent: (10)	4 (40)	5 (50)	0 (0)	3 (30)
Minimal: (15)	8 (53)	9 (60)	8 (53)	11 (73)
Moderate: (9)	4 (44)	5 (56)	8 (89)	6 (67)
**Cirrhosis**				
Present:12 (35)	6 (50)	6 (50)	6(50)	9 (75) #
Absent: 22 (65)	10 (45%)	13 (59)	10(45)	11 (50)
**Hepatitis grade**				
I &II: (26)	11 (42)	12 (46)	9 (35)	15 (58)
III&IV: (8)	5 (63)	7 (88) #	7 (88) #	5 (63)
**Hepatitis stage**				
I &II: (25)	12 (48)	13 (52)	8 (32)	16 (64)
III &IV: (9)	4 (44)	6 (67)	8 (89) #	4 (44)

Bcl-xL was not expressed in any of the studied CH cases. # significantly difference (p < 0.005).

## Discussion

An important cause of morbidity and mortality worldwide is the infection by HCV. Progress in understanding HCV biology has remained challenging due to the lack of an efficient cell culture system for virus growth. Establishment of self-replicating full-length HCV genomic replicons from genotypes in cultured cells has provided an important tool for the study of HCV replication mechanisms. This study discusses the system for the HepG2 cell line harboring HCV- genotype-4 replication and examines the expression levels of group of genes in clinical samples obtained from HCC and CH patients. Other studies have reported another systems for HCV replication, the first with HCV GT1 H77 in immortalized human hepatocytes (IHH) [34] and the other system of HCV GT2 JFH1 in human hepatoma cell line (Huh7) [35]. Kanda *et al. *suggested that IHH support HCV genome replication and virus assembly by examined HCV core protein-mediated IHH for growth of HCV [34]. Their study described the generation of cell culture-grown HCV from genotype 1a and discuss the concept of HCV replication and assembly of genotype 1a in IHH and speculated that cellular defense mechanisms against HCV infection are attenuated or compromised in IHH [34]. It was reported the HCV production from a HCV-ribozyme construct of genotype 1a (clone H77) in Huh-7 cells with no determination for the virus infectivity [35]. Furthermore, subgenomic replicons of the JFH1 genotype 2a strain cloned from an individual with fulminant hepatitis replicate efficiently in cell culture. The JFH1 genome replicates efficiently and supports secretion of viral particles after transfection into a Huh7, providing a powerful tool for studying the viral life cycle and developing antiviral strategies [35].

Apoptosis has been demonstrated as an important mechanism for viral clearance. In HCV-infected liver, viral persistence is observed despite enhanced hepatocyte apoptosis [5]; however, it is not clear whether this apoptotic effect is due to a direct cytopathic effect of the virus, immunological reactions or a contribution of the molecular mechanisms causing liver damage during HCV infection [22,36]. For understanding the impact of HCV infection on the apoptotic machinery during disease progression, we studied the expression patterns of Bcl-2, Bcl-xL, Bak, Fas, FasL in HCV- genotype-4 infected HepG2 cell line as well as in human tissue samples obtained from patients with HCC and CH as a result of chronic HCV infection. We also analyzed the expression levels of caspases 3, 8 and 9 in tissue culture medium and in HCV infected cells by a colorimetric assay, and viral replication by both RT-PCR and Real-Time PCR for up to 135 days post-infection.

The results of the present study showed that HCV infection disrupted the process of apoptosis through down regulation of Fas and up-regulation of FasL genes expression. However, in tissue samples a higher expression of Fas and FasL genes were detected in CH compared to HCC patients, which explains the presence of severe inflammation in chronic HCV infection and its oncogenic potential. In this regard, previous studies demonstrated that enhanced FasL gene expression induces T-cell apoptosis [15], which favors viral persistence and indirectly increases the probability of progression to HCC [36]. In addition, the FasL gene exerts proinflammatory activities via IL-1β secretion that is responsible for neutrophils infiltration [37].

In contrast, other studies [38-40] demonstrated that the ratio of Fas/FasL was significantly lower in HCC than in CH tissue samples or non tumor hepatic tissues. This was attributed to the fact that tumor cells possess more than one safe guard against Fas mediated apoptosis. First, the reduced expression or loss of certain molecules that are involved in the Fas mediated apoptosis pathway such as FADD (Fas-associated protein with death domain), FLICE (FADD like interleukin-1β-coverting enzyme, caspase-8) or FAF (Fas associated factor), or the induction of molecules that would inhibit Fas mediated apoptosis such as FAP (Fas associated phosphatase) [7]. Second, the expression of sFas RNA and FAP-1 may neutralize Fas mediated apoptosis [41] and third, Fas mutation could be expected. Many investigators suggested that one of the possible mechanisms by which HCV core protein inhibits apoptosis is through a direct binding to downstream domain of FADD and cFLIP leads to viral persistence and cells proliferation [5]. Consequently, it is conceivably possible that the observed decreased apoptosis relative to cell proliferation of infected hepatocytes could be part of the signaling mechanisms in the pathogenesis of HCC [42].

It has also been reported that the extrinsic (Fas-FasL) pathway plays an important role in liver cell injury directly via HCV infection or indirectly through immune attack of HCV- infected cells with subsequent recruitment and activation of stellate cells and macrophages, resulting in fibrosis and cirrhosis [43]. Also, I was found that during HCV infection, HCV-specific T cells migrate to the liver and recognize viral antigens on the hepatocytes [38]. These immunologically active cells, which are probably induced due to inflammation rather than viral infection, become activated and express FasL that transduces the apoptotic death signal to *Fas *bearing hepatocytes, resulting in their destruction [38]. Therefore, neither *Fas *expression nor the degree of liver injury correlates with the intra-hepatic viral load [15,44]. In such case, the TNF or the IFN-δ might be responsible for the up regulation of Fas expression in infected hepatocytes and FasL in lymphocytes [45].

Alternatively, the hepatocytes which are likely type II cells in which direct activation of caspase 8 (extrinsic pathway mechanism) is not sufficient to induce apoptosis amplification by a mitochondrial pathway (intrinsic mechanism) are highly required. Accordingly caspase 8 activation causes the proapoptotic cleavage of *Bid*, which induces *cytochrome c *release from the mitochondria, which subsequently binds to Apaf-1 and procaspase 9 forming apoptosome complex [29]. In the present study, we assessed the activation of caspases 8, and 9, which represent both death receptor-mediated and the mitochondrial apoptosis pathway and caspase 3 which is an executioner caspase. Our data showed a positive correlation between Fas mediated apoptosis and caspases activation. In HCV infected cells, we observed a loss of caspases after 4 weeks post HCV infection. Some studies provided evidence that monitoring of caspases activation might be helpful as a diagnostic tool to detect the degree of HCV mediated inflammatory liver damage and to evaluate efficacy of HCV therapy [36,37]. However, it was reported that the extent of caspase activation correlates with the grade of the disease but not with surrogate markers, such as serum transaminases or viral load [36]. This observation indicates that caspase activation is not directly related to HCV mediated damage and suggests the involvement of HCV mediated immune response with Fas triggered hepatocyte apoptosis giving rise to several amplification loops [36]. Similar findings were reported by others, who indicated in their study that the core protein could stimulate caspase-independent apoptosis at later stages of the disease giving relevance to the release of HCV particles from the host cells and to viral spread [46].

It has been shown that some HCCs are resistant to Fas-mediated apoptosis directly through the expression of HCV proteins or indirectly through up-regulation of Bcl-2 family members [36]. Our data showed that both Bcl-2 and Bcl-xL RNA expression were significantly higher in HCC than in CH and NDT indicating late involvement of those genes in the cascade of HCV-associated hepatocarcinogenesis. We were also able to detect Bcl-2 gene expression in HepG2 cells starting from day 1 post-infection until the end of the experiment, whereas the expression of Bcl-xL was not visible until day 28 when it started to be expressed and its expression was closely associated with the presence of HCV in tumor cells (Table 3) suggesting that Bcl-2 is tumor related whereas Bcl-xL is a viral related. In this context, Bcl-2 was linked to inhibition of apoptosis via interfering with either the recruitment of procaspase 8 to Fas receptors [47] or by preventing the release of cytochrome C [5]. It has also been shown that the HCV core protein inhibits apoptosis at the mitochondrial level through augmentation of Bcl-xL expression with consequent inhibition of caspase 3 activation [16]. The HCV core protein could induce apoptosis in the Fas death way although this is achieved through the activation of Bax and Bak, both are important mediators of p53 mitochondrial function [5,36]. Our results showed an increase in Bak-RNA expression at an early stage of HCV infection of HepG2 cells, which is also observed in tissue samples obtained from both CH and HCC patients compared to NDT samples. Our results provided enough evidence that the Bak gene can induce apoptosis in HCC cells even in the presence of high levels of the anti-apoptotic Bcl-2 gene family members, which is in agreement with the findings of others [48].

The results of gene expression in tissue samples show a significant correlation between Fas expression in HCC cases and the presence of cirrhosis or poorly differentiated tumors. We observed that FasL expression was significantly associated in CH patients with the grade of inflammation and the stage of fibrosis as well as with the presence of severe necro-inflammatory changes. Based on these results we conclude that aberrant expression of Fas and FasL in HCV-infected patients could be considered a marker for increased disease severity with a higher possibility of progression into cirrhosis and/or HCC. Similar results were also reported by others [49], who indicated that FasL may contribute to malignant transformation of hepatocyte as it was significantly expressed in the peri-cancerous lobules and cirrhotic nodules [42]. Similarly, Bcl-2 expression was significantly associated with poorly-differentiated tumors as well as with the presence of cirrhosis in CH patients. Similar findings were reported previously by some of us [32]. In this study, Bak expression was significantly associated with absence of cirrhosis and well-differentiated tumors, thus Bak gene could be considered a good prognostic marker.

The impact of HCV infection on modulating apoptotic machinery pathway(s) differs during the course of infection, as the disease progresses apoptosis is inhibited leading to cell immortalization and HCC development. HCV infection could exert a direct effect on hepatocytes by inducing Fas-FasL pathway with subsequent inactivation of caspases or indirectly by immune attack on hepatocytes resulting in HCV mediated liver injury, viral persistence and cirrhosis in CH patients with an increasing possibility of hepatocarcinogenesis especially with increasing proliferation rate and acquisition of genetic damage.

Alternatively, HCV infection could induce apoptosis at the early phase of infection followed by modulation of apoptosis by disturbing Fas/FasL. This in turn would cause an inactivation of caspases 3, 8, and 9, up-regulation of Bcl-2 family members, impairment in Bak gene expression and increasing the expression of FasL leading to inhibition of apoptosis in HCV infected patients. This signaling cascade favors cell survival with persistence of HCV infection and enhances the possibility of HCC development. A combination of these effects initiates a circle of hepatocyte damage and repair, which is the hallmark of HCV infection that might progress to HCC. Our study could provide an insight for understanding apoptosis and developing molecular target therapies that could inhibit viral persistence and HCC development. Further studies are still required to clarify the interaction between other HCV proteins in the apoptotic machinery system and the possible involvement of other apoptotic pathways in HCV associated HCC development.

## Conclusions

Chronic HCV infection modulates the apoptotic machinery differently during the course of infection, where the virus induces apoptosis early in the course of infection, and as the disease progresses apoptosis is modulated. This study could open a new opportunity for understanding the various signallings of apoptosis and in the developing a targeted therapy to inhibit viral persistence and HCC development. Nevertheless, further studies are mandatory to clarify the interaction between other HCV proteins in the apoptotic machinery system and the possible involvement of other apoptotic pathways in HCV associated HCC development.

## Competing interests

The authors declare that they have no competing interests.

## Authors' contributions

ARNZ made substantial contributions to conception and design, carried out the tissue culture and molecular genetic studies and gave the final approval of the version to be published. AAB carried out pathological and the immunohistochemistry studies. MMH carried out the tissue culture and molecular genetic studies, participated in the design of the study and performed the statistical analysis. ZKH participated in the molecular studies and participated in the statistical analysis, interpretation of data and drafted the manuscript. MK participated in pathological studies. SAL participated in drafting the manuscript. GMS participated in the statistical analysis. AREZ provided all clinical samples and data. SSD participated in drafting the manuscript and revised the manuscript critically for important intellectual content. All authors read and approved the final manuscript.
